# Comparison of continuous 24‑hour and 14‑day ECG monitoring for the detection of cardiac arrhythmias in patients with ischemic stroke or syncope

**DOI:** 10.1002/clc.24247

**Published:** 2024-03-07

**Authors:** Wei‐Cheng Chen, Yu‐Lin Wu, Yu‐Cheng Hsu, Jen‐Te Hsu, Hung‐Pin Tseng, Chao‐Chin Chen, Meng‐Hsiu Chiang, Ju‐Feng Hsiao, See‐Khong Chin, Ying‐Li Huang, Meng‐Huan Lei

**Affiliations:** ^1^ Division of Cardiology, Department of Internal Medicine Lo‐Tung Poh‐Ai Hospital Luodong Taiwan; ^2^ Post‐Baccalaureate Program in Nursing, College of Nursing Taipei Medical University Taipei Taiwan; ^3^ Division of Neurology, Department of Internal Medicine Lo‐Tung Poh‐Ai Hospital Luodong Taiwan

**Keywords:** 14‐day ECG patch, 24‐h Holter, atrial fibrillation, cardiac arrhythmias, stroke, syncope

## Abstract

**Background:**

Previous studies show that using 12‐lead electrocardiogram (ECG) or 24‐h ECG monitor for the detection of cardiac arrhythmia events in patients with stroke or syncope is ineffective.

**Hypothesis:**

The 14‐day continuous ECG patch has higher detection rates of arrhythmias compared with conventional 24‐h ECG monitoring in patients with ischemic stroke or syncope.

**Methods:**

This cross‐sectional study of patients with newly diagnosed ischemic stroke or syncope received a 24‐h ECG monitoring and 14‐day continuous cardiac monitoring patch and the arrhythmia events were measured.

**Results:**

This study enrolled 83 patients with ischemic stroke or syncope. The detection rate of composite cardiac arrhythmias was significantly higher for the 14‐day ECG patch than 24‐h Holter monitor (69.9% vs. 21.7%, *p* = .006). In patients with ischemic stroke, the detection rates of cardiac arrhythmias were 63.4% for supraventricular tachycardia (SVT), 7% for ventricular tachycardia (VT), 5.6% for atrial fibrillation (AF), 4.2% for atrioventricular block (AVB), and 1.4% for pause by 14‐day ECG patch, respectively. The significant difference in arrhythmic detection rates were found for SVT (45.8%), AF (6%), pause (1.2%), AVB (2.4%), and VT (9.6%) by 14‐day ECG patch but not by 24‐h Holter monitor in patients with ischemic stroke or syncope.

**Conclusions:**

A 14‐day ECG patch can be used on patients with ischemic stroke or syncope for the early detection of AF or other cardiac arrhythmia events. The patch can be helpful for physicians in planning medical or mechanical interventions of patients with ischemic stroke and occult AF.

## Graphical abstract

- This study compared the performance of 14‐day continuous ECG patch with conventional 24‐h ECG monitoring for the detection of arrhythmias in 83 patients with ischemic stroke or syncope.
- The detection rate of composite cardiac arrhythmias was significantly higher for the 14‐day ECG patch than 24‐h Holter monitor (69.9% vs. 21.7%, *p* = .006). In patients with ischemic stroke, the detection rates of cardiac arrhythmias were 63.4% for supraventricular tachycardia (SVT), 7% for ventricular tachycardia (VT), 5.6% for atrial fibrillation (AF), 4.2% for atrioventricular block (AVB), and 1.4% for pause by 14‐day ECG patch, respectively.
- A 14‐day ECG patch can be used on patients with ischemic stroke or syncope for the early detection of AF or other cardiac arrhythmia events. The patch can be helpful for physicians in planning medical or mechanical interventions of patients with ischemic stroke and occult AF.

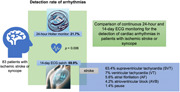

**Abbreviations**
AF: atrial fibrillation
APCs: atrial premature complexes
AVB: atrioventricular block
ECG: electrocardiogram
ED: emergency department
OAC: oral anticoagulant
SVT: supraventricular tachycardia
VF: ventricular fibrillation
VT: ventricular tachycardia

## 1 INTRODUCTION

Cardiac arrhythmias include atrial fibrillation (AF), ventricular tachycardia (VT), ventricular fibrillation (VF), supraventricular tachycardia (SVT), sinus bradycardia/pauses, and atrioventricular block (AVB).^1^ Cardiac arrhythmias cause various symptoms, such as palpitation, dizziness, chest tightness, and syncope; they can lead to sudden cardiac death in highly serious cases^2^ and affect the patient's quality of life.^3^

AF was detected in 15% of cases of stroke in older patients who had a cryptogenic transient ischemic attack and ischemic stroke.^4^ A systematic review reported that the prevalence of AF has been observed to be from 0.37% to 3.56% in hospital‐based studies and 2.8% to 15.8% in community‐based studies in Asian countries.^5^ Jauch et al.^6^ found that the prevalence of AF is 0.1% in patients aged <55 years and 10% in patients aged >80 years. AF is associated with ischemic stroke,^5^, ^7^ heart failure,^8^ and systemic embolization.^9^ In addition, cardiac arrhythmia was detected in 21.9% of patients with ischemic stroke by a 24‐h electrocardiogram (ECG) system.^10^

Syncope is a symptom in patients with arrhythmia^11^ and defined as a relatively brief and self‐limited transient loss of consciousness.^12^ Syncope is a risk factor for sudden cardiac arrest in patients with coronary artery disease.^13^ A previous study demonstrated that 35% of patients who presented to emergency department (ED) with syncope had serious arrhythmia, which was detected using 12‐lead ECG, within 30 days from the index ED visit (the median time to diagnosis was 2 days [interquartile range was 1–5 days]).^14^ Because the standard 12‐lead ECG has only a 10‐s strip, 12‐lead ECG is not an effective measurement for detecting cardiac arrhythmias in patients with stroke or syncope. 

By contrast, 24‐h ECG monitor, which is also called Holter monitor, can increase detection rate of cardiac arrhythmias; however, the wearable ECG can interfere with patients' life activities.^15^ A wearable and wireless 14‐day ECG can continuously record a patient's heartbeat up to 14 days and has higher detection rate of cardiac arrhythmias than 24‐h ECG monitor.^16^, ^17^, ^18^, ^19^

In some patients with stroke or syncope, cardiac arrhythmias cannot be identified using 12‐lead ECG or 24‐h ECG monitor, which delays effective treatment. A previous study suggests that a 14‐day monitoring period is more favorable for detecting arrhythmias.^20^ Therefore, this study compared the detection rates between 24‐h ECG monitor and 14‐day ECG patch and determined the association between the CHA_2_DS_2_–VASc score and cardiac arrhythmias in patients with stroke or syncope.

## 2 MATERIALS AND METHODS

This cross‐sectional study investigated patients with ischemic stroke or syncope who were examined using a 24‐h Holter ECG monitoring and 14‐day continuous cardiac monitoring patch (Ezypro, Sigknow Biomedical). This study was approved by China Medical University and Hospital Research Ethics Committee (No. CMUH108‐REC2‐030).

We enrolled patients from March 2019 to December 2021. The following patients were included: (1) patients diagnosed with ischemic stroke or syncope for the first time and (2) patients aged >20 years. The following patients were excluded: (1) patients with ischemic stroke related to lacunar infarcts, large cerebral artery disease, or cardiac emboli; (2) patients with positive tilt‐table test; (3) patients with arrhythmia history; (4) patients who received beta blockers, nondihydropyridine CCB, amiodarone, propafenone, antiarrhythmic agents, and other Class I, III antiarrhythmic agents; (5) patients with skin allergies or skin diseases; and (6) pregnant women.

### 2.1 Study protocols and devices

Physicians contacted eligible patients, and all enrolled patients provided their written informed consent. Patients received both 24‐h ECG monitor and a 14‐day ECG patch simultaneously or within 3 days after diagnosing ischemic stroke or syncope.

### 2.2 Arrhythmic events and score of stroke risk

Arrhythmic events were measured for SVT (>4 beats),^20^ AF (>30 s),^20^ pauses (>3 s),^20^ AVB (second‐degree, 2:1, or third‐degree AV block, as confirmed by cardiologists),^20^ VT (>4 beats),^20^ and VF. A composite cardiac arrhythmia was defined by the presence of anyone of the measured arrhythmias. Each patient's stroke risk was estimated using the CHA_2_DS_2_–VASc score, a point‐based system. In this system, 1 point is assigned for each of the following: being aged between 65 and 74 years; having a history of hypertension or receiving antihypertensive agents, having a history of diabetes or receiving insulin or oral hypoglycemic agents, having a history of congestive heart failure, having a history of myocardial infarction or peripheral artery disease, and being a woman. Moreover, in the system, 2 points are assigned for each of the following: being aged ≥75 years, having a history of stroke, and having a history of transient ischemic attack.^21^ Medical records were reviewed to confirm comorbidities.

### 2.3 Data analysis

Data analysis was performed using SAS software 9.2 version. The mean, percentage, and variance were calculated. Fisher's exact test was used to compare the 24‐h Holter monitor and 14‐day ECG patch with respect to their detection rate of cardiac arrhythmia events. The Mann–Whitney *U* test was applied to test the difference in CHA_2_DS_2_–VASc between patients with and without cardiac arrhythmias.

## 3 RESULTS

### 3.1 Participant demographics

In total, 83 patients met the inclusion criteria. Table 1 shows patient demographics and clinical characteristics. The average age of patients was 60.3 (*SD* = 13) years. Among these patients, 70 participants had a diagnosis of stroke (84.3%), and 13 participants had a diagnosis of syncope (15.7%). The mean of the CHA_2_DS_2_–VASc score was 3.4 (*SD* = 1.2).

**Table 1:** Baseline characteristics of patients (*N* = 83).

	Total (*N* = 83)	Stroke (*N* = 70)	Syncope (*N* = 13)	
	Mean (*SD*)	Mean (*SD*)	Mean (*SD*)	Mann–Whitney *U* test
Age (years)	60.3 (13.0)	59.9 (11.3)	60.2 (20.2)	0.517
SBP (mmHg)	139.3 (23.1)	142.0 (23.7)	124.6 (12.8)	0.012
DBP (mmHg)	84.5 (15.9)	86.5 (15.9)	75.2 (10.6)	0.006
HR (bpm)	68.5 (9.9)	67.9 (9.3)	72.9 (12.7)	0.118
Echocardiography				
LA (mm)	35.4 (5.8)	35.8 (5.5)	32.3 (7.9)	0.148
E/A ratio	0.88 (0.4)	0.84 (0.3)	1.2 (0.7)	0.349
E/e'	10.7 (4.4)	10.8 (4.4)	8.8 (5.8)	0.647
CHA2DS2‐VASc score	3.4 (1.2)	3.59 (1.04)	2.42 (1.56)	0.016

**Past history**	***N* (%)**	***N* (%)**	***N* (%)**	**Fisher's exact test**
Hypertension	52 (62.7%)	43 (61.4%)	8 (61.5%)	0.475
Hyperlipidemia	24 (28.9%)	20 (28.6%)	4 (30.8%)	0.477
DM	17 (20.5%)	16 (22.9%)	1 (7.7%)	0.239
CAD	1 (1.2%)	1 (1.4)	0 (0)	0.855
CKD	4 (4.8%)	2 (2.9%)	2 (15.4%)	0.098

*Note*: E/A ratio: ratio between early (E) and late (atrial—A) ventricular filling velocity; E/e': ratio of transmittal early filling velocity over early diastolic tissue velocity.

Abbreviations: CAD, coronary artery disease; CKD, chronic kidney disease; DM, diabetes mellitus; LA, left atrial.

### 3.2 Wearing duration and complication

All patients completed the monitoring using the 24‐h Holter monitor and 14‐day ECG patch. The mean wearing duration (proportion of available duration for analysis) was 22 h (80%) for the 24‐h Holter monitor and 12.5 days (90%) for the 14‐day ECG patch. Notably, three patients (3.6%) reported skin rashes at the site of 14‐day ECG patch attachment.

### 3.3 Detection rate of cardiac arrhythmias

Table 2 shows the detection rate of cardiac arrhythmias for the 14‐day ECG patch and 24‐h Holter monitor. The detection rates of SVT and AVB were significantly higher for the 14‐day ECG patch than 24‐h Holter monitor (*p* = .007; *p* = .002). Table 3 demonstrates the cardiac arrhythmias events, namely SVT, AF, pause, AVB, and VT, were detected in 38 (45.8%), 5 (6%), 1 (1.2%), 2 (2.4%), and 8 (9.6%) patients, respectively, using 14‐day ECG patch but not by 24‐h Holter monitor.

**Table 2:** Detection rate for cardiac arrhythmia events of 14‐day ECG patch and 24‐h Holter monitor (*N* = 83).

	Total events/detection rate (%)		*p* value (Fisher's exact test)
	14‐day ECG patch	24‐h Holter monitor	
CCA	58 (69.9%)	18 (21.7%)	.006
SVT	53 (64%)	15 (18.1%)	.007
AF	5 (6.0%)	0	–
pause	1 (1.2%)	0	–
AVB	4 (4.8%)	2 (2.4%)	.002
VT	9 (10.8%)	1 (1.2%)	.108
VF	0	0	–

Abbreviations: AbbCCA, composite cardiac arrhythmia; AF, atrial fibrillation; AVB, atrioventricular block; ECG, electrocardiogram; SVT, supraventricular tachycardia; VF, ventricular fibrillation; VT, ventricular tachycardia.

**Table 3:** Cardiac arrhythmia events detected using 14‐day Ezypro and 24‐h Holter.

Cardiac arrhythmias	E+H+	E+H−	E−H+	E−H−
SVT	15 (18.1%)	38 (45.8%)	0	30 (36.1%)
AF	0	5 (6%)	0	78 (94.0%)
pause	0	1 (1.2%)	0	82 (98.8%)
AVB	2 (2.4%)	2 (2.4%)	0	79 (95.2%)
VT	1 (1.2%)	8 (9.6%)	0	74 (89.2%)
VF	0	0	0	83 (100%)

*Note*: E: Ezypro; H: Holter; +: detected cardiac arrhythmia event; −: did not detect cardiac arrhythmia event.

Abbreviations: AF, atrial fibrillation; AVB, atrioventricular block; SVT, supraventricular tachycardia; VF, ventricular fibrillation; VT, ventricular tachycardia.

**Figure 1:**
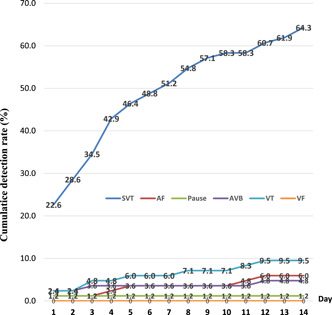
Cumulative detection rate of cardiac arrhythmias by the 14‐day ECG patch. ECG, electrocardiogram.

More composite cardiac arrhythmias were detected in 58 (69.9%) patients using the 14‐day ECG patch than in 18 (21.7%) patients using the 24‐h Holter monitor (*p* = .006). Composite cardiac arrhythmias were detected in 47 (67.1%) and 11 (84.6%) patients with stroke and syncope, respectively, by the 14‐day ECG patch and in 16 (22.9%) and 2 (15.4%) patients with stroke and syncope, respectively, by the 24‐h Holter monitor. Table 4 shows the detection rates for cardiac arrhythmias, namely SVT, AF, pause, AVB, VT, and VF in patients with stroke and syncope using the 14‐day ECG patch and 24‐h Holter monitor. The significant differences of CCA detection rate between 14‐day ECG patch and 24‐h Holter monitor were found in overall participants (*p* = .006) and both subgroups for diagnosis by stroke and syncope (both *p* < .001). Figure 1 shows the daily cumulative detection rate for cardiac arrhythmias of the 14‐day ECG patch.

**Table 4:** Detection rate of arrhythmias using 14‐day ECG patch and 24‐h monitor in patients with stroke and syncope.

	Total *N*	Detection rate %	*p* a value	Stroke (*N* = 70)			Syncope (*N* = 13)		
				*N*	Detection rate %	*p* a value	*N*	Detection rate %	*p* a value
**CCA**									
14‐day ECG patch	58	69.9	.006	47	67.1	<.001	11	84.6	.004
24‐h monitor	18	21.7		16	22.9		2	15.4	–
**SVT**									
14‐day ECG patch	53	63.9	.007	44	62.9	.001	9	69.2	–
24‐h monitor	15	18.1		15	21.1		0		–
**AF**									
14‐day ECG patch	5	6.0	–	4	5.6	–	1	7.7	–
24‐h monitor	0	–	–	0	–		0		–
**pause**									
14‐day ECG patch	1	1.2	–	1	1.4	–	0	–	–
24‐h monitor	0	–	–	0	–	–	0	–	–
**AVB**									
14‐day ECG patch	4	4.8	.002	3	4.2	.042	1	7.7	.830
24‐h monitor	2	2.4		1	2.8		1	7.7	
**VT**									
14‐day ECG patch	9	10.8	.108	5	7.0	–	4	30.8	.333
24‐h monitor	1	1.2		0	–	–	1	7.7	
**VF**									
14‐day ECG patch	0	–	–	0	–	–	–	–	–
24‐h monitor	0	–	–	0	–	–	–	–	–

Abbreviations: AF, atrial fibrillation; AVB, atrioventricular block; CCA, composite cardiac arrhythmia; ECG, electrocardiogram; SVT, supraventricular tachycardia; VF, ventricular fibrillation; VT, ventricular tachycardia.

^a^ Fisher's exact test.

### 3.4 Risk of stroke scores (CHA_2_DS_2_–VASc)

Patients with SVT or composite cardiac arrhythmias had significantly higher CHA_2_DS_2_–VASc scores (median = 4.0, 25%–75% = 3–5) than patients without SVT (median = 3.0, 25%–75% = 2.75–3.3) (*p* = .004). For the composite cardiac arrhythmias, significantly higher CHA_2_DS_2_–VASc score was found in patient with composite cardiac arrhythmias (median = 4.0, 25%–75% = 3–4.3) than patients without composite cardiac arrhythmias (median = 3.0, 25%–75% = 3–3.3) (*p* = .024).

## 4 DISCUSSION

The findings showed that the detection rate of composite cardiac arrhythmias was significantly higher for the 14‐day ECG patch than 24‐h Holter monitor (69.9% vs. 21.7%). In addition, 1.2%–45.8% of cardiac arrhythmia events, namely those of SVT, AF, pause, AVB, and VT, were detected only by the 14‐day ECG patch but not detected by 24‐h Holter monitor in patients with stroke and syncope. Therefore, a 14‐day ECG patch is a useful tool for detecting arrhythmia events. The findings of this study on the detection rates of cardiac arrhythmia events are comparable to those of a previous study, where 66% of patients were diagnosed with paroxysmal arrhythmia using a 14‐day ECG patch, whereas 9% of patients were diagnosed with that condition by a 24‐h Holter monitor.^19^ A previous study on silent AF for patients with coronary disease, heart failure, hypertension, diabetes, and sleep apnea showed that AF presented in 5.3% of patients and atrial tachycardia presented in 67% of patients as detected using a 14‐day ECG patch.^22^

Cardiac thromboembolism that is attributed to AF accounted for one‐third of cases of ischemic stroke.^23^ In patients with stroke, new‐onset ECG abnormalities, cardiac arrhythmias, and ventricular repolarization abnormalities were present in 75%, 28.7%, and 14.2% of patients, respectively.^10^ However, in older patients with high risk of stroke (CHA_2_DS_2_–VASc score >1), the detection rate of AF was 4% for the 14‐day ECG patch and 1.1% for the 24‐h Holter monitor.^21^ An open‐label randomized controlled trial of cardiac monitoring after ischemic stroke or transient ischemic attack showed that the detection rate of AF at 28 days was 14.0% in the patch‐based 14‐day monitoring group, as compared to 2.1% in the Holter monitoring groups (*p* = .05).^24^ In our study, we found that the detection rate of AF was 5.6% for the 14‐day ECG patch and 0% for the 24‐h Holter monitor. A previous study showed that the cardiac arrhythmia of atrial premature complexes and SVT using 24‐h Holter were significant predictors of stroke 11 years later.^25^ A study reported that patients with screen‐detected AF without oral anticoagulant (OAC) had highest stroke risk as compared with patients without AF. The risk of stroke in patients with screen‐detected AF was comparable to clinically diagnosed AF and could be reduced by OAC use.^26^ Our study demonstrated that the detection rates of composite arrhythmias, namely SVT, AF, pause, AVB, VT, and VF, were three times higher by the 14‐day ECG patch as compared with the 24‐h Holter monitor (67.1% vs. 22.9%) in patients with ischemic stroke. The longer period of ECG monitoring for composite arrhythmias would increase the accuracy of clinical diagnoses and improve the treatment strategies for patients with ischemic stroke.

An international, multicenter, prospective trial evaluate the role of external 4‐week ECG monitoring in clinical work‐up of unexplained syncope with suspected arrhythmic origin. They showed that the 4‐week diagnostic yield was 24.5% and early start of recording 10–15 days versus >15 days after index event was significant predictor of diagnostic events (odds ratio: 6.2). Accordingly, they suggested that the 4‐week external ECG monitoring can be considered as an effective first‐line tool in the diagnostic work‐up of syncope.^27^ In our study, 84.6% of patients with syncope were detected with composite arrhythmias using 14‐day ECG patch, and 15.4% of patients were diagnosed with composite arrhythmias using 24‐h Holter monitor. Therefore, patients with syncope should be monitored for longer periods to detect the composite arrhythmias in future clinical practices.

A previous study reported that 15% of patients with suspected arrhythmia‐related symptoms were diagnosed with arrhythmia using 14‐day ECG patch, and the detection rate of VT significantly differed between patients with CHA_2_DS_2_–VASc scores of <3 versus ≥4.^20^ Another study on Japanese patients aged >65 years and with CHA_2_DS_2_–VASc scores higher than 1 found that 4% of patients had AF that was detected using 14‐day external loop monitoring, and only 1.1% of patients had AF that was detected using 24‐h Holter monitor.^21^ In patients with ischemic stroke or syncope, this study found the CHA_2_DS_2_–VASc scores in patients with SVT or CCA events (median 4) were significantly higher than patients without any arrhythmia events (median 3). Therefore, physicians should use ECG with longer monitoring period for detecting arrhythmia in patients with a higher risk of stroke that is assessed using CHA_2_DS_2_–VASc score (≥4).

A longer period of ECG monitoring is recommended for clinical use for increasing the detection rate of arrhythmia. However, the side effect of ECG patch, such as skin irritation need to be considered.^17^ In a previous study on 25 751 patients, the mean wear time of 14‐day Zio Patch ECG monitor was 7.6 days, and the median analysis time was 99% of the total wear time.^28^ In addition, in another study, 8.9% of patients with cryptogenic stroke were found to have AF by 6 months, which was detected using insertable cardiac monitor.^29^ In our study, the detection rate of AF was 6% in patients with stroke or syncope. A longer period of ECG monitoring can increase the detection rate of cardiac arrhythmia; however, the exact duration of this period needs to be assessed in additional studies to formulate more sensitive methods of detecting cardiac arrhythmia with less side effects.

Regarding costs, a 14‐day ECG patch costs US$270, and a 7‐day ECG patch costs US$175. Using the 14‐day ECG patch for long‐term monitoring resulted in a relatively high detection rate for arrhythmias. Patients can choose between the 7‐ and 14‐day ECG patch options depending on their economic circumstances. In general, both options are considered affordable and feasible.

Apart from cost considerations, detecting clinically relevant arrhythmias such as AF through ECG monitoring carries crucial clinical implications. Upon the detection of such arrhythmias, anticoagulant drugs may be prescribed to patients to prevent stroke recurrence.

### 4.1 Study limitations

This study has several limitations. The sample size of patients with ischemic syncope was relatively small in this study. Ischemic stroke was presumed to be cryptogenic after evaluation with 12‐lead ECG, 24‐h Holter monitor, transthoracic echocardiography, ultrasonography of cervical artery, brain computed tomography scan and magnetic resonance imaging. Screening for thrombophilic state and transesophageal echocardiography were not performed mostly.

## 5 CONCLUSIONS

A 14‐day ECG patch can be used on patients with ischemic stroke or syncope for the early detection of arterial fibrillation or other cardiac arrhythmia events. The patch can be helpful for physicians for planning medical or mechanical interventions of patients with ischemic stroke and occult AF.

## CONFLICT OF INTEREST STATEMENT

The authors declare no conflicts of interest.

## Data Availability

Research data are not shared.
